# Evolving pituitary hormone deficits in primarily isolated GHD: a review and experts’ consensus

**DOI:** 10.1186/s40348-020-00108-2

**Published:** 2020-11-03

**Authors:** Gerhard Binder, Dirk Schnabel, Thomas Reinehr, Roland Pfäffle, Helmuth-Günther Dörr, Markus Bettendorf, Berthold Hauffa, Joachim Woelfle

**Affiliations:** 1 University Children’s Hospital, Pediatric Endocrinology, Hoppe-Seyler-Str. 1, 72076 Tübingen, Germany; 2 Center for Chronic Sick Children, Pediatric Endocrinology, Charité, University Medicine Berlin, Berlin, Germany; 3 Vestische Children’s Hospital, Pediatric Endocrinology, Diabetes and Nutrition Medicine, University of Witten/Herdecke, 45711 Datteln, Germany; 4 University Children’s Hospital Leipzig, Pediatric Endocrinology, University of Leipzig, Liebigstr. 20a, 04103 Leipzig, Germany; 5 University Children’s Hospital, Pediatric Endocrinology, 91301 Erlangen, Germany; 6 Division of Paediatric Endocrinology and Diabetes, University Children’s Hospital Heidelberg, Im Neuenheimer Feld 430, 69120 Heidelberg, Germany; 7 University Children’s Hospital, Pediatric Endocrinology, University of Duisburg-Essen, Hufelandstr. 55, 45122 Essen, Germany; 8 University Children’s Hospital, Pediatric Endocrinology, Loschgestr. 15, 91054 Erlangen, Germany

**Keywords:** Isolated GHD, Combined pituitary hormone deficiency, Organic GHD, Pituitary gland malformation, Acquired GHD

## Abstract

Isolated growth hormone deficiency (GHD) is defined by growth failure in combination with retarded bone age, low serum insulin-like growth factor-1, and insufficient GH peaks in two independent GH stimulation tests. Congenital GHD can present at any age and can be associated with significant malformations of the pituitary-hypothalamic region or the midline of the brain. In rare instances, genetic analysis reveals germline mutations of transcription factors involved in embryogenesis of the pituitary gland and the hypothalamus. Acquired GHD is caused by radiation, inflammation, or tumor growth. In contrast to organic GHD, idiopathic forms are more frequent and remain unexplained.

There is a risk of progression from isolated GHD to combined pituitary hormone deficiency (> 5% for the total group), which is clearly increased in children with organic GHD, especially with significant malformation of the pituitary gland. Therefore, it is prudent to exclude additional pituitary hormone deficiencies in the follow-up of children with isolated GHD by clinical and radiological observations and endocrine baseline tests. In contrast to primary disorders of endocrine glands, secondary deficiency is frequently milder in its clinical manifestation. The pituitary hormone deficiencies can develop over time from mild insufficiency to severe deficiency. This review summarizes the current knowledge on diagnostics and therapy of additional pituitary hormone deficits occurring during rhGH treatment in children initially diagnosed with isolated GHD. Although risk factors are known, there are no absolute criteria enabling exclusion of children without any risk of progress to combined pituitary hormone deficiency. Lifelong monitoring of the endocrine function of the pituitary gland is recommended in humans with organic GHD. This paper is the essence of a workshop of pediatric endocrinologists who screened the literature for evidence with respect to evolving pituitary deficits in initially isolated GHD, their diagnosis and treatment.

## Background

Childhood-onset growth hormone deficiency (GHD) is frequently occurring as an isolated hormone deficiency [1], but in a subgroup of these children, additional pituitary hormone deficiencies evolve over time. This development implies either a genetically determined vulnerability or masked insufficiency of pituitary or hypothalamic cells or an acquired damage of the hypothalamic-pituitary axis with progressive dysfunction. Treatment with recombinant human GH (rhGH) may unmask TSH deficiency in some patients with GHD [2]. Additional pituitary deficits also arise over time in monogenic hypopituitarism caused by mutations in *PROP1*, *POU1F1*, *HESX1*, and other developmental genes [3]. Acquired GHD due to suprasellar tumor, cranial irradiation, or other causes of damage is accompanied or followed by deficits of other pituitary hormones, which frequently evolve in a characteristic pattern [4]. Therefore, the diagnosis of GHD may implicate the potential rise of additional pituitary hormone deficits.

There is no consensus on how to monitor and identify those patients with GHD in need for treatment with additional hormones and which diagnostic and prognostic markers are informative. The reported prevalence of evolving pituitary deficits in GHD clearly depends on the study inclusion criteria (idiopathic versus organic GHD), the duration of follow-up, and the clinical criteria used for the indication of dynamic testing. It seems rational to assume a higher likelihood of upcoming additional pituitary hormone deficiencies in children with monogenic disorders, with developmental disorders of the pituitary gland and the midline, after cranial irradiation, and with an inflammatory process or tumor in the hypothalamic-pituitary region. This paper is the essence of a workshop of pediatric endocrinologists who screened the literature for evidence with respect to evolving pituitary deficits in initially isolated GHD, their diagnosis and treatment.

### Prognostic markers

Large long-term follow-up studies evaluating major risk factors that predict the occurrence of additional pituitary hormone deficiencies in isolated GHD are missing. The largest study with this target evaluating data from the Genesis study (*n* = 5805) had a relatively short median follow-up of 4 years in a subgroup with 1757 patients with an incidence of combined pituitary hormone deficiency (CPHD) of 5.5% [5]. An analysis of the literature on prognostic risk factors for the progression from isolated GHD to CPHD was recently reported by Cerbone and Dattani [4]. Accordingly, children with organic GHD etiology are more likely to develop CPHD than those with idiopathic GHD. Especially, children with structural abnormalities of the pituitary gland and the brain midline like absent pituitary stalk, ectopic posterior pituitary, abnormal corpus callosum, empty sella, and septo-optic dysplasia spectrum are prone to develop CPHD. Similarly, the relatively rare genetic defects in transcription factors such as HESX1, PROP1, POU1F1, LHX3, LHX4, SOX2, and SOX3 are associated with variable phenotypes (some only presenting with isolated GHD) and frequently associated with the development of CPHD. This is even true for children with the dominantly inherited isolated GHD type 2 with splice site mutations of GH1, who may develop additional pituitary hormone deficiencies in rare instances [6]. Similarly, acquired damage of the hypothalamic-pituitary axis is very likely to proceed from isolated GHD to multiple pituitary hormone deficiency [7], and in specific damages like the post-radiation condition, prediction is possible based on the dose of radiation [8]. The severity of isolated GHD may also be a hint to additional hormone deficiencies evolving over time [5]. In contrast, children with partial GHD or mild idiopathic GHD are rarely affected by additional pituitary hormone deficiencies.

Therefore, it seems reasonable to estimate the risk for developing combined pituitary hormone deficiency individually and to judge clinical and laboratory data based on this specific risk estimate. Moderately reduced serum hormone concentrations should be controlled earlier in organic GHD than in idiopathic GHD. Stimulatory tests for the detection of additional pituitary hormone deficits should earlier be performed in organic GHD than in idiopathic GHD. Such timing would enable early detection of patients in need of additional hormone therapy and also avoid false positives, which may be falsely substituted with hormones.

Nevertheless, it has to be stressed that the diagnosis of idiopathic isolated GHD does not exclude the occurrence of additional hormone deficiencies in the future implicating a thorough follow-up of the pituitary axes in all children with isolated GHD.

### Diagnosis of TSH deficiency

Thyroid-stimulating hormone (TSH) deficiency is the most frequent secondary pituitary hormone deficiency in children initially diagnosed with GHD, with a reported prevalence of 6.3% during the first 2 years of treatment [2]. The clinical characteristics of TSH deficiency are milder than in primary hypothyroidism. An insufficient increase of IGF-1 and a low growth response to rhGH treatment have been suggested as possible signs of evolving TSH deficiency [9], but this has not been studied systematically. The ultrasound of the thyroid gland may be normal in TSH deficiency at diagnosis. After 1-year substitutional treatment of children and adolescents with TSH deficiency, the volume of the thyroid gland did not change [10].

The laboratory confirmation of TSH deficiency is done by consecutive measurements of basal serum fT4 which stay below the reference interval in the presence of a low-normal to moderately elevated serum TSH concentration [11]. The TRH challenge is not reliable, does not provide additional information, and should not be used [11, 12]. TSH deficiency should be distinguished from the frequently and early occurring decrease of fT4 during the first year of rhGH treatment, which is probably a reflection of an enhanced conversion of thyroxine to triiodothyronine and associated with a rise of fT3 [13, 14]. The consecutive fT4 nadir is transient and disappears after 6 months of rhGH treatment in the majority of treated children [10, 15]. However, it must be stressed that both TSH deficiency and enhanced conversion of thyroxine to triiodothyronine can cause fT4 serum levels below the reference interval after 3 months on rhGH. In the absence of a significant malformation of the pituitary gland or other historical, clinical, or genetic findings implying high likelihood of the occurrence of TSH deficiency, the experts considered it rational to wait and control fT4 3-monthly before treatment with l-thyroxine is initiated. The exception is any very low fT4 concentration that is unlikely to be transient.

### Treatment of TSH deficiency

The treatment with l-thyroxine can cause a life-threatening adrenal crisis in patients with untreated ACTH deficiency [12, 16]. Therefore, hypocortisolism should be excluded before thyroid hormone replacement. In the absence of randomized trials, experts recommend a low l-thyroxine starting dose (i.e., 25 to 50 μg/day total) that should be increased weekly to reach the target of around 1.5 μg/kg day (for school children) within approx. 1 month. The main aim is a serum fT4 concentration in the upper normal range (> 50th percentile) accompanied by a normal fT3. Experts’ opinions are heterogeneous with respect to TSH serum values [12, 17]; most emphasize the invalidity of this parameter in the presence of TSH deficiency. Nevertheless, it must be stressed that suppressed TSH is a very frequent finding in well-treated children with TSH deficiency and certainly in the majority of cases not a sign of overdosing. In children with GHD and TSH deficiency on rhGH, the introduction of l-thyroxine treatment was shown to increase resting energy expenditure and shorten the myocardial isovolumic contraction time, which was correlated to total T3, but not total T4 [18]. It seems rational to reevaluate the thyroid axis by a drug holiday for 4 weeks, especially if GHD is not confirmed in adolescence.

### Diagnosis of ACTH deficiency

Adrenocorticotropic hormone (ACTH) deficiency rarely occurs as an evolving pituitary deficit after the diagnosis of isolated GHD, and frequently, it is the last one becoming deficient. Reported prevalence ranges from less than 1 to 12%, with higher numbers in children with concomitant TSH deficiency or pituitary malformations [5, 19, 20]. ACTH deficiency has been described in patients with hypopituitarism caused by *PROP1*, *POU1F1*, *LHX3*, *LHX4*, and *GLI2* mutations. In these instances, the diagnosis was frequently made at the initial presentation. Rarely, ACTH deficiency has been reported in children with autosomal dominant GHD due to *GH1* mutations [6].

ACTH deficiency can evolve in acquired hypopituitarism after cranial irradiation > 30 Gy [21], sellar and suprasellar tumors, hemochromatosis, sarcoidosis, Langerhans cell histiocytosis and after tumor surgery. Here, indeed, hypopituitarism may start with isolated GHD and then proceed to CPHD including ACTH deficiency. Central hypocortisolism causes the same signs and symptoms as Addison’s disease, but in contrast to primary hypocortisolism, it is characterized by normal or hypopigmented skin and normal mineralocorticoid reserve. In addition, the clinical phenotype may be milder and adrenal crisis at the time of diagnosis less frequent than in primary adrenal insufficiency [22]. Routine monitoring of adrenal sufficiency by measuring morning serum cortisol may minimize this risk in children with organic GHD.

The diagnosis of ACTH deficiency should be based on testing and clinical suspicion, which may be raised by failure to thrive, loss of appetite, loss of weight, hypopigmentation, low blood pressure, dizziness, decreased vitality, and other findings. The recommended baseline test is measurement of the morning cortisol [23]. Morning serum cortisol concentrations > 275 nmol/l are considered to exclude ACTH deficiency, and concentrations < 85 nmol/l confirm ACTH deficiency [23]. DHEAS production of the adrenal gland is also ACTH dependent. Whereas the measurement of serum DHEAS is a good baseline test in postpubertal adolescents, this is not the case in younger children and adolescents with still rising DHEAS production [24].

In the case of borderline low morning cortisol values (< 275, but > 85 nmol/l) and clinical suspicion, a short Synacthen test is recommended. There is still discussion about the best way to this test. Some experts recommended the low-dose Synacthen test (1 μg i.v. absolute) because of increased accuracy [25]. Others prefer the standard Synacthen test (125 μg or 250 μg i.v. absolute) claiming that the exact administration of 1 μg Synacthen by diluting it from an ampoule containing 250 μg may be technically challenging, even as Synacthen sticks to plastic and glass surfaces [26]. A systematic review and meta-analysis did not detect an advantage of the low-dose versus the high-dose Synacthen dose [27]. Recommended cutoffs vary. Kazlauskaite and Maghnie recommended for both tests a stimulated serum cortisol concentration > 490 nmol/l for exclusion and < 360 nmol/l for confirmation of ACTH deficiency [23]. In dubious cases with values in between, an additional insulin-hypoglycemia test (ITT) or glucagon test is recommended. The ITT cutoff is 490 or 550 nmol/l [23, 28]. The ITT is potentially dangerous, and its use should be restricted to older children.

Sometimes, hormone substitution may precipitate ACTH deficiency: treatment with rhGH attenuates 11β-HSD-type-1 isoenzyme activity, thereby leading to reduced cortisone-to-cortisol conversion [29]. Treatment with l-thyroxine can unmask ACTH deficiency and provoke adrenal crisis [16].

### Treatment of ACTH deficiency

Oral hydrocortisone is the first choice of treatment of secondary adrenal insufficiency in childhood. The recommended dose is 8–10 mg/m^2^ BSA TID daily [30]. Traditionally, used hydrocortisone doses are lower than in primary adrenal insufficiency [31]. Residual ACTH secretion, concomitant therapies, and diseases may influence the replacement dose; patients with hypopituitarism on treatment with rhGH and estrogens may need higher glucocorticoid dose as compared to patients in whom GHD and hypogonadism are not replaced [32].

The strategy of personalized dosing as well as the teaching of the patient and caregivers regarding stress-dose and emergency glucocorticoid administration does not differ from primary adrenal insufficiency. However, published guidelines for children do not exist [33]. In female adolescents with central adrenal insufficiency that suffer from depression or lack of libido, the additional hormone replacement with 25 mg DHEA per day is beneficial according to a randomized controlled trial [34].

The incidence of adrenal crisis in individuals with adrenal insufficiency when treated is approximately 5–10 events per 100 patient years in adults [35]. The risk of adrenal crisis seems to be lower in secondary than in primary adrenal insufficiency, but pediatric data are lacking [36]. Death in children monitored during rhGH treatment could be traced back to adrenal crisis related to ACTH deficiency in 12–25% of the cases [37].

There are no good biochemical markers for monitoring glucocorticoid replacement in patients with ACTH deficiency. Glucocorticoid replacement should be based on the general dose recommendations, well-being and physical vitality of the patient, physiological increase in body weight, normal fat distribution, normal height velocity, and normal blood pressure as suggested for patients with primary adrenal insufficiency [38]. The hydrocortisone dose should be kept as low as possible.

### Diagnosis of gonadotropin deficiency

Gonadotropin deficiency is the second most frequent pituitary hormone deficiency evolving in children with isolated GHD; the estimated prevalence ranges from 4.5 to 38% [20, 39]. However, as gonadotropin deficiency is clinically silent in childhood (at least in girls), estimates based on cohorts of children of any age are likely underestimations. Some hypothalamic syndromes like Prader-Willi syndrome (PWS) are characterized by frequent combination of both GH and gonadotropin deficiency [40]. Mutations of the developmental gene *SOX2* can cause anophthalmia, esophageal atresia, and genital tract abnormalities associated with GH and gonadotropin deficiency [41]. The most frequent monogenic defect of pituitary development with recessive inheritance is caused by *PROP1* mutations, which is characterized by the combination of GH, TSH, ACTH, and gonadotropin deficiency [42].

Micropenis and bilateral cryptorchidism are important clinical clues in male infants. The earliest window of opportunity to diagnose gonadotropin deficiency by hormone measurements is minipuberty during the first 4 to 6 months of life [43] when sex hormones and gonadotropins reach midpubertal serum levels in both genders during this time period [44]. There are examples for dissociation between minipuberty and puberty in certain disorders (Prader-Willi syndrome, hypogonadotropic hypogonadism due to KISS1R loss of function) that must be kept in mind [45].

At pubertal age, the manifestation of gonadotropin deficiency comprises the broad spectrum from no entry into puberty to an uncompleted or prolonged puberty. In adolescents with IGHD, the differential diagnosis of delayed puberty between constitutional delay in growth and puberty (CDGP) or hypogonadotropic hypogonadism can be difficult. In the presence of idiopathic IGHD and a positive family history for CDGP, the likelihood of gonadotropin deficiency is low. The likelihood increases in organic isolated GHD, in the absence of a positive family history for CDGP, and in boys with a history of bilateral orchidopexy or micropenis. The distinction on biochemical parameters includes the baseline measurement of inhibin B, LH, FSH, and sex hormones [46]. Reference intervals and cutoffs, however, heavily rely on the assays used: therefore, one should prefer immunoassays or liquid chromatography-tandem MS assays with adequate reference data for the pediatric population. The most accurate dynamic test is the GnRH agonist challenge with an LH cutoff at 4 h being approx. 5 IU/l in adolescent boys [47]. For girls, data are sparse. According to a recent small study, stimulated FSH after GnRH challenge is more discriminatory than LH in adolescent girls; the cutoff was 11 IU/l [48]. In adolescents with isolated GHD, it is crucial to diagnose gonadotropin deficiency as early as possible, as treatment should be offered when the peer group goes into puberty.

### Treatment of gonadotropin deficiency

The choice of drug in girls with gonadotropin deficiency is estradiol. In a recent international consensus statement, transdermal estradiol substitution was preferably recommended started at low doses (0.06 μg/kg nocturnally) and increased slowly over 24 months to ensure normal breast development. Estradiol dosing is thereby aiming to mimic estradiol levels during gonadarche. A typical target dose in adulthood would be 50 μg estradiol daily by patch, or one to two pumps of 0.06% estradiol gel [49]. Subsequently, cyclic progestin should be added for 14 days. The pleaded advantages in comparison to oral treatment are minimization of the first path effect, higher IGF-1 serum levels [50], better feminization [51], and potentially a better cardiovascular risk profile in adulthood [52, 53]. Major disadvantages are the lack of suitable estradiol patches, the lack of long-term trials comparing transdermal and oral treatment, the lack of knowledge with respect to adherence, and the absence of approval for the transdermal indication. In a recent paper, Donaldson et al. offered detailed instructions how the recommended low estradiol doses can be reached by cutting the available estradiol patches into small pieces and proposed a protocol for estradiol dosing during a 3-year pubertal induction [54]. Fertility treatment is successful in the majority of women with hypogonadotropic hypogonadism [55].

In male hypogonadotropic hypogonadism, the induction of puberty is classically achieved with intramuscular depot testosterone (testosterone enanthate). The alternative is the combined subcutaneous administration of HCG and rhFSH enabling almost normal testicular growth and timely spermatogenesis [56]. Both treatments can be offered also during minipuberty in boys with micropenis due to hypogonadotropic hypogonadism [57]. Puberty induction by testosterone enanthate is based on long clinical practice and associated with high adherence, but inhibits testicular growth and spermatogenesis. Induction of puberty by gonadotropins promotes spermatogenesis and testicular volume, but is dependent on good adherence and lacks long clinical experience. It remains to be proven whether a higher rate and an earlier onset of fertility in adulthood can be achieved by puberty induction using gonadotropins, but evidence is missing yet. Predictors of reduced fertility in male hypogonadotropic hypogonadism are inborn cryptorchidism as well as low testicular volume and low inhibin B serum levels at the start of puberty induction [58].

### Diagnosis of ADH deficiency

Anti-diuretic hormone (ADH) deficiency very rarely evolves after the diagnosis of isolated GHD. The highest prevalence reported in a Brazilian group of children with severe GHD was 5% [20]; the lowest 0.2% in a big cohort with strictly defined isolated GHD from the Genesis observational trial [5]. Probably, the more frequent pattern of evolution is ADH deficiency followed by GH deficiency [59]. In the presence of GHD, ADH deficiency may be associated with septo-optic dysplasia and other severe midline defects such as holoprosencephaly. More frequently, pituitary and hypothalamic surgery, infundibulitis, craniopharyngioma, germinoma, histiocytosis, severe brain trauma, or acute brain insults are causative [60]. Therefore, the occurrence of diabetes insipidus in children with isolated GHD should always arouse suspicion of the presence of histiocytosis, germinoma, or infundibulitis, which all can cause GHD. In such children, a lesion of the pituitary stalk and the pituitary gland should be excluded by cranial magnetic resonance imaging (cMRI) with gadolinium contrast [61].

The main clinical symptoms are polyuria and polydipsia, which should systematically be monitored after exclusion of diabetes mellitus and hypercalciuria. The definition of polydipsia varies; a fluid intake > 2 l/m^2^ per day is the most common one [62]. Adrenal insufficiency and hypothyroidism can mask ADH deficiency. Traditionally, complete ADH deficiency is confirmed by insufficient increase in urinary molarity at the end of a water deprivation test (< 300 mosm/kg) and a significant increase of urinary molarity after subsequent administration of desmopressin (> 750 mosm/kg). A marker of ADH secretion is copeptin, the C-terminal portion of the precursor peptide of arginine vasopressin, which is secreted in equimolar amounts from the posterior pituitary and more stable in serum. First experiences in a pediatric cohort, however, attest a relatively low specificity at 67% for the distinction from primary polydipsia [63].

A triphasic pattern in the disturbance of ADH secretion, which is characterized by diabetes insipidus and syndrome of inappropriate ADH secretion (SIADH), may occur after neurosurgery in the hypothalamic-pituitary region. A transient postoperative polyuria is followed by oliguria and then often permanent polyuria [64].

### Treatment of ADH deficiency

If ADH deficiency cannot be compensated by moderately increased fluid intake or causes severe sleep disturbance, the synthetic ADH analog desmopressin is the treatment of choice. Administration routes are multiple; the preferred one is orally, as water intoxication and hyponatremia occur less likely than after intranasal administration [65, 66]. The bioavailability of the intranasal desmopressin administration is ten times less than the intravenous administration, and ten times more than the oral administration. The new desmopressin lyophilisate for oral absorption is approximately 1.7 times more potent than the conventional tablet. Overdosing of desmopressin and/or excessive fluid intake independent of thirst can cause water intoxication and potentially life-threatening brain edema [65]. Therefore, teaching of the patient and caregivers regarding individual fluid intake and situational adaptation of the desmopressin dose is paramount. Interactions of other drugs with endogenous ADH secretion and tubular action need to be considered [65].

## Conclusions

Treatment of children with isolated GHD demands regular monitoring of the other pituitary hormone axes, because over time isolated GHD can progress to CPHD. This dynamic can be slow, and its detection is mainly based on the measurement of pituitary and peripheral hormones (Table 1). Relevant risk factors for this development are organic GHD, especially significant malformations of the pituitary-hypothalamic region, and acquired GHD. The manifestation of TSH deficiency is most frequent, and ADH deficiency is rare. Treatment is based on the experience made with peripheral gland disorders. However, treatment monitoring should abstain from the conventional judgment of the pituitary hormone level, because this one is deficient. Lifelong monitoring of the pituitary gland function is recommended in humans with organic GHD.

**Table 1:** Endocrine investigations for monitoring children with isolated GHD

Pituitary hormone	Baseline investigation	Second-line investigation
TSH	fT4, TSH	Not needed
ACTH	Morning cortisol (7–9 am)	Synacthen test, insulin-hypoglycemia test
LH/FSH	Inhibin B, LH, FSH, testosterone/estradiol	GnRH agonist test
ADH	Early morning serum/urine osmolality	Water deprivation test, desmopressin test

## Data Availability

Not applicable.

## Abbreviations

GHD: Growth hormone deficiency
CPHD: Combined pituitary hormone deficiency
rhGH: Recombinant human GH
TSH: Thyroid-stimulating hormone
ACTH: Adrenocorticotropic hormone
ITT: Insulin-hypoglycemia test
CDGP: Constitutional delay in growth and puberty
ADH: Anti-diuretic hormone
cMRI: Cranial magnetic resonance imaging
SIADH: Syndrome of inappropriate ADH secretion
